# Supplementary material for: The Complex Relationship Between Serum Uric Acid, Endothelial Function and Small Vessel Remodeling in Humans
Source: J Clin Med. 2020 Jun 28;9(7):2027. doi: 10.3390/jcm9072027 (PMC7409082; doi:10.3390/jcm9072027)

SUPPLEMENTARY MATERIAL

The complex relationship between serum uric acid, endothelial function and small vessel remodeling in humans

METHODS

Population

Levels of serum uric acid (SUA) were available in three (Brescia, Pisa, Milano) of the four centres (Brescia, Pisa, Milano, Rome) contributing to the constitution of the microvascular dataset of the Italian Society of Hypertension(1, 2), corresponding to a total of 162 subjects with information on SUA levels and M/L ratio. All subjects with information on SUA had data on traditional cardiovascular risk factors included in the European Heart SCORE. In 70 of these subjects, measures of endothelial function and NO availability were available in the Pisa's dataset, in which the pressurised myographic technique and biopsies taken from the anterior abdominal wall were used for all experiments. The dataset inclusion criteria were age >18 years and the availability of microvascular remodeling parameters (M/L ratio). Subjects with a history of previous cardiovascular (CV) events or presence of chronic kidney disease stage 4-5 were excluded (1, 2).

Microvascular parameters

To estimate the media-to-lumen ratio, the thickness of the media and the diameter of the lumen were measured in three-to-six different points for each small artery. These measures were subsequently averaged to calculate the M/L ratio. The media cross-sectional area (MCSA) was obtained by subtracting the internal from the external cross-sectional area (CSA) that, in turn, were calculated using the lumen and external vascular diameters, respectively.

To define the predominant pattern of small vessel remodeling, the growth and remodeling indexes were calculated according with the Baumbach and Heistad formula(3):

Remodelling Index: IntDRef – (ExtDtest2 – 4 x MCSARef / π)1/2 / (IntDRef – IntDtest)

Growth Index: (MCSAtest - MCSARef) / MCSARef

where IntD refers to the internal and ExtD refers to the external vessel diameter of the reference group (IntDRef and ExtDRef) or the test (ExtDRef and ExtDTest) groups, while MCSA refers to the media cross-sectional area of the reference (MCSARef) and test (MCSATest) groups (3). The measure of growth index was available in 118 subjects, while the remodeling index was available in 72 subjects.

After acquisition of the structural parameters, the endothelial function was assessed by dose-response curves to acetylcholine (ACh 0.001–100 μM) in vessels precontracted with norepinephrine (1 μM)(2). To estimate the proportion of endothelial dysfunction due to NO availability, the dose-response curves to acetylcholine were repeated after a 30-min incubation with the endothelial NO synthase inhibitor L-NAME (100 μM). Thus, the NO availability was calculated by subtracting the maximal vasodilation obtained with acetylcholine plus L-NAME from that obtained with acetylcholine alone (2, 4).

References

1. Bruno RM, Grassi G, Seravalle G, Savoia C, Rizzoni D, Virdis A, et al. Age- and Sex-Specific Reference Values for Media/Lumen Ratio in Small Arteries and Relationship With Risk Factors. Hypertension. 2018;71(6):1193-200.
2. Masi S, Georgiopoulos G, Chiriaco M, Grassi G, Seravalle G, Savoia C, et al. The importance of endothelial dysfunction in resistance artery remodelling and cardiovascular risk. Cardiovascular research. 2020;116(2):429-37.
3. Heagerty AM, Aalkjaer C, Bund SJ, Korsgaard N, Mulvany MJ. Small artery structure in hypertension. Dual processes of remodeling and growth. Hypertension. 1993;21(4):391-7.
4. Bruno RM, Duranti E, Ippolito C, Segnani C, Bernardini N, Di Candio G, et al. Different Impact of Essential Hypertension on Structural and Functional Age-Related Vascular Changes. Hypertension. 2017;69(1):71-8.

SUPPLEMENTARY TABLES

Table 1S. The linear and non-linear relationship between SUA and parameters of microvascular remodeling in males and females.

	Males				Females			
	**Linear term**		**Non-linear term**		**Linear term**		**Non-linear term**	
	β (95% CI)	P value	β (95% CI)	P value	β (95% CI)	P value	β (95% CI)	P value
M/L ratio	-0.870 (-1.44, -0.305)	0.003	0.323 (0.122, 0.525)	0.002	-0.014 (-0.025, -0.003)	0.014	0.01 (0.002, 0.012)	0.014
MCSA	-0.400 (-0.74, -0.06)	0.022	0.35 (0.01, 0.061)	0.009	-0.895 (-1.34, -0.453)	<0.001	0.075 (0.035, 0.115)	<0.001

M/L ratio and MCSA variables were transformed with the natural logarithm

Table 2S. The linear and non-linear relationship between SUA and parameters of microvascular remodeling in subjects with and without arterial hypertension.

	Population with normal blood pressure				Population with Hypertension			
	**Linear term**		**Non-linear term**		**Linear term**		**Non-linear term**	
	β (95% CI)	P value	β (95% CI)	P value	β (95% CI)	P value	β (95% CI)	P value
M/L ratio	-0.327 (-0.629, -0.024)	0.035	0.027 (0.003, 0.051)	0.025	-0.011 (-0.018 -0.004)	0.003	0.005 (0.002, 0.008)	0.003
MCSA	-5.039 (-8.017, -2.061)	0.001	1.107 (0.483, 1.730)	0.001	-0.018 (-0.027, -0.009)	<0.001	0.008 (0.004, 0.012)	<0.001

M/L ratio and MCSA variables were transformed with the natural logarithm

Table 3S. The linear and non-linear relationship between SUA and parameters of microvascular remodeling/endothelial function in subjects with eGFR > 60 ml/min.

**M/L ratio**				**MCSA**				**Endothelial function**		**NO Availability**	
**Linear term**		**Non Linear term**		**Linear term**		**Non Linear term**					
Coefficient (95% CI)	P-value	Coefficient (95% CI)	P value	Coefficient (95% CI)	P value	Coefficient (95% CI)	P value	Coefficient (95% CI)	P value	Coefficient (95% CI)	P value
-0.386 (-0.602, -0.173)	<0.001	0.03 (0.016, 0.05)	<0.001	-1.71 (-2.41, -1.01)	<0.001	0.623 (0.373, 0.874)	<0.001	-4.83 (-7.97, -1.69)	0.003	-5.69 (-8.49,-2.88)	<0.001

M/L ratio and MCSA variables were transformed with the natural logarithm

Table 4S. Differences in the relationship of growth and remodeling indexes among microvascular assessment methods

	Growth Index				Remodeling Index			
	Linear term		Non-Linear term		Linear term		Non-Linear term	
	Coefficient (95% CI)	P-value	Coefficient (95% CI)	P value	Coefficient (95% CI)	P value	Coefficient (95% CI)	P value
**Overall**	7.1 (1.7, 12.42)	0.01	-6.78 (-12.2, -1.42)	0.014	0.734 (0.113, 1.35)	0.021	-0.05 (-0.104, -0.002)	0.041
**Myographic technique**								
Pressure-myography	0.015 (0.004, 0.025)	0.007	-0.01 (-0.011, -0.002)	0.01	0.014 (-0.007, 0.035)	0.179	0.01 (-0.015, 0.003)	0.185
Wire-myography	7.6 (1.63, 13.5)	0.014	-7.9 (-14.2, -1.54)	0.016	-10.5 (-16.5, 37.4)	0.429	26.7 (-65.1, 11.63)	0.163

SUPPLEMENTARY FIGURES

Supplementary Figure 1. Diagram of the attrition of the population included in the SIIA adaset based on the availability of data for each analysis. Abbreviations: HS = Heart Score.

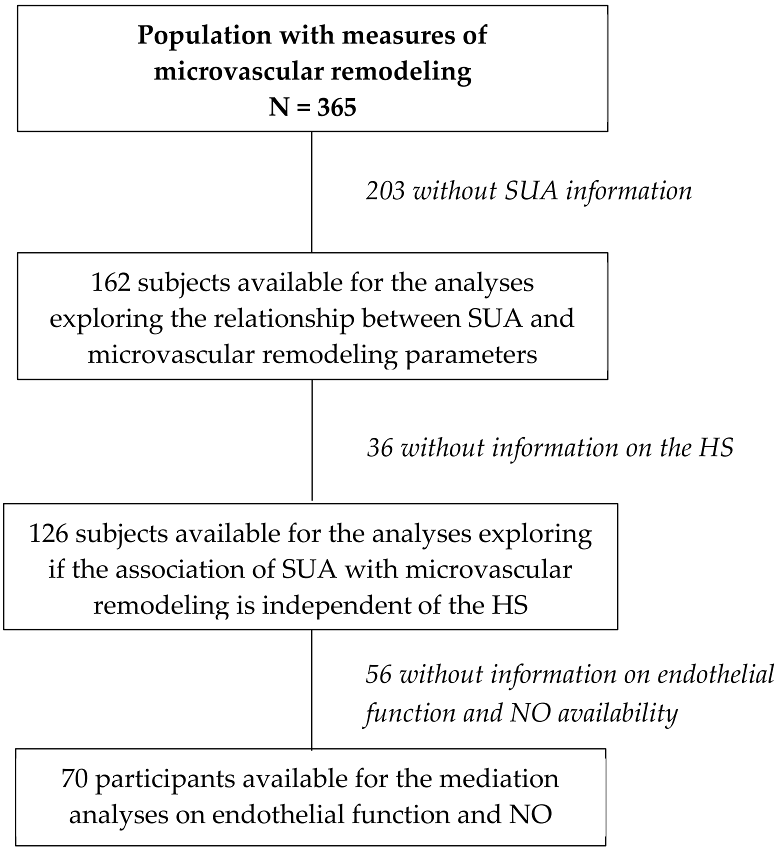

Supplementary Figure 2. Fractional polynomial regression analysis for the association between continuous levels of serum uric acid (SUA) and **A)** growth index (n=118) and **B)** remodeling index (n=72). Serum uric acid is modelled as a second-degree fractional polynomial.

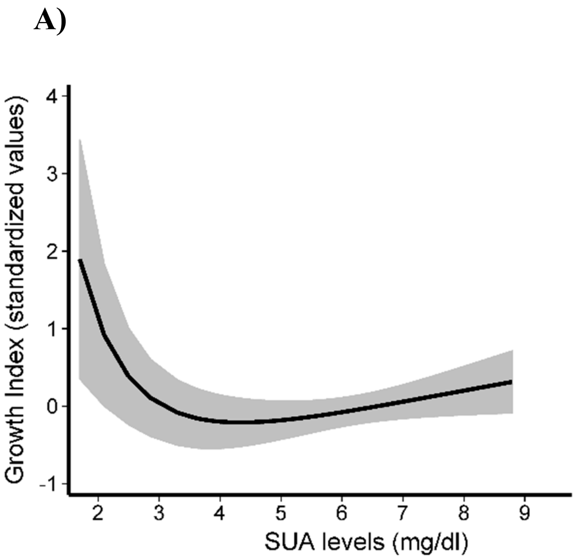

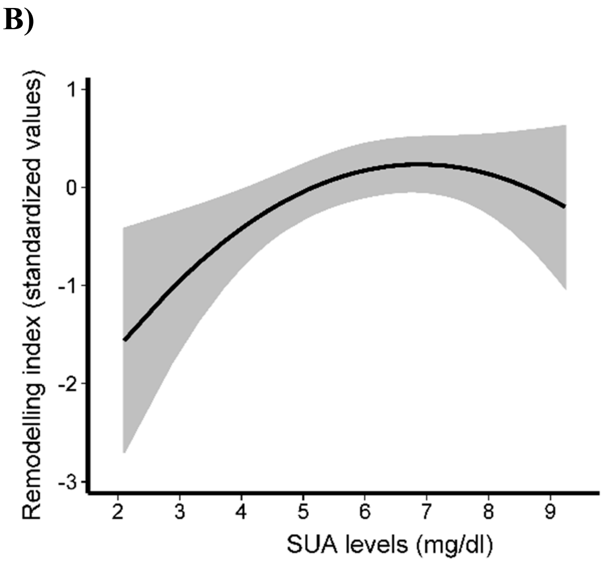

Supplement: Supplementary file 1 [file jcm-09-02027-s001.pdf]
